# Evaluation of the Azotofertil Biostimulant as a Nitrogen-Based Fertilization Strategy for *Solanum lycopersicum* L. and *Capsicum annuum* L.

**DOI:** 10.3390/plants15162475

**Published:** 2026-08-15

**Authors:** Alina Elena Marta, Ștefănica Stoica, Mihaela Covașă, Carmenica Doina Jităreanu

**Affiliations:** 1 Department of Plant Science, Faculty of Agriculture, “Ion Ionescu de la Brad” Iasi University of Life Sciences, 3 Mihail Sadoveanu Alley, 700489 Iasi, Romania; alina.marta@iuls.ro (A.E.M.);; 2 Department of Pedotechnics, Faculty of Agriculture, “Ion Ionescu de la Brad” Iasi University of Life Sciences, 3 Mihail Sadoveanu Alley, 700489 Iasi, Romania

**Keywords:** microbial biostimulant, Azotobacter chroococcum, *Azospirillum lipoferum*, tomato, pepper, total yield, vitamin C, total chlorophyll

## Abstract

The continuous growth of the global population and the need to ensure food security require the development of sustainable agricultural technologies capable of maintaining crop productivity while reducing environmental impact. Nitrogen-fixing bacteria belonging to the genera *Azotobacter* and *Azospirillum* have attracted increasing interest due to their ability to improve plant nutrition and stimulate physiological processes associated with growth and productivity. The aim of the present study was to evaluate the effect of the commercial biostimulant Azotofertil, based on *Azotobacter chroococcum* and *Azospirillum lipoferum*, on the agrophysiological response of tomato and pepper plants, in comparison with conventional nitrogen and phosphorus fertilization. A field experiment was conducted using a randomized block design with three fertilization treatments and three biological replicates for each crop species. Soil pH, cultivable *Azotobacter* spp. populations, plant height, photosynthetic pigment content, vitamin C content, and total yield were evaluated. Data were analyzed using one-way analysis of variance (ANOVA), followed by Duncan’s multiple range test (*p* < 0.05) and Pearson correlation analysis. The application of Azotofertil significantly increased the abundance of cultivable *Azotobacter* spp., photosynthetic pigment content, and vitamin C content, while no statistically significant differences were observed for soil pH or plant height. The most pronounced effect was observed for photosynthetic pigment content, which increased by 26% in tomato and by 21% in pepper compared to the control.

## 1. Introduction

The continuous growth of the global population, estimated to exceed 9 billion people by mid-century, places increasing pressure on agricultural systems, requiring the development of sustainable strategies for enhancing plant production, given that plants represent the primary source of food for both the human population and the livestock sector [1,2]. Within crop production, horticultural plants occupy a particularly important place due to their high nutritional value and significant contribution to a balanced diet. Fruits and vegetables are essential sources of vitamins, minerals, dietary fiber, and bioactive compounds with beneficial effects on human health, which is why there is a growing interest in expanding and improving their cultivation [3,4].

Tomatoes (*Solanum lycopersicum* L.) are among the most important and widely cultivated vegetable species worldwide, due to their high nutritional, economic, and agronomic value. They are appreciated both for their rich content of vitamins, minerals, and bioactive compounds with antioxidant properties, and for their adaptability to various cultivation systems, as well as for their significant share in global horticultural production [5,6,7]. Pepper (*Capsicum annuum* L.) is also among the most widely cultivated vegetable species worldwide, holding significant nutritional and economic importance. Its popularity is determined by its high nutritional value, rich content of vitamins and antioxidant compounds, as well as its extensive use both in fresh consumption and in the food industry [8,9,10,11].

Adequate mineral nutrition is a key factor determining crop productivity, and among essential elements, nitrogen plays a central role due to its involvement in numerous physiological and biochemical processes, directly influencing vegetative growth, photosynthesis, and biomass accumulation [12,13,14]. However, the efficiency of nitrogen fertilization is often reduced by significant nitrogen losses from the soil, caused by processes such as leaching, volatilization, and denitrification, which lead both to a reduction in nitrogen availability for plants and to negative environmental impacts [15,16].

Nitrogen-fixing bacteria have attracted considerable interest in modern agriculture due to their ability to supply nitrogen to plants through biological nitrogen fixation and to promote plant growth through various mechanisms, including phytohormone production and enhanced nutrient uptake [17,18]. Bacteria of the genus *Azotobacter* are among the most widely used nitrogen-fixing microorganisms in agriculture, due to their ability to improve nitrogen availability for plants. In addition, they contribute to plant growth stimulation through the production of phytohormones and other beneficial metabolites, which is why they are frequently included in biostimulant formulations aimed at increasing productivity and nutrient use efficiency [19,20,21]. Species of the genus *Azospirillum* are associative nitrogen-fixing bacteria capable of colonizing the soil and contributing to improved plant nitrogen nutrition. Moreover, they enhance root system development and increase the efficiency of water and nutrient uptake, which is why they are frequently used in biostimulant formulations, often in combination with *Azotobacter* species to achieve complementary effects on crop growth and productivity [22,23].

The productivity and quality of horticultural crops are closely dependent on growth conditions and plant physiological status, aspects that can be assessed through relevant biochemical parameters. Chlorophyll content represents an indicator of photosynthetic capacity and plant nutritional status, while the content of antioxidant compounds, particularly vitamin C, reflects the nutritional value of the yield and the plants’ ability to respond to various stress factors [24,25,26,27,28].

Although microbial biostimulants based on nitrogen-fixing bacteria have been extensively investigated, their performance may vary depending on crop species, environmental conditions, soil characteristics, and the composition of the microbial consortium. Therefore, evaluating the agronomic and physiological responses of economically important horticultural crops under specific cultivation conditions remains necessary to better understand their effectiveness in comparison with conventional fertilization strategies.

Therefore, the aim of the present study was to evaluate the effects of a commercial microbial biostimulant containing *Azotobacter chroococcum* and *Azospirillum lipoferum* on the growth, physiological performance, fruit quality, and yield of tomato (*Solanum lycopersicum* L.) and pepper (*Capsicum annuum* L.). The purpose of this approach is not to completely replace conventional fertilization or to achieve superior performance, but rather to evaluate the potential of *Azotobacter chroococcum* and *Azospirillum lipoferum*, applied through a commercial biostimulant formulation, to support crop yield and quality at levels comparable to those achieved through conventional fertilization, while reducing mineral nitrogen inputs and improving nitrogen use efficiency.

## 2. Results and Discussion

### 2.1. Soil pH Analysis

The analysis of soil pH revealed slight changes in its values following the application of the two fertilizer treatments. However, none of these differences were statistically significant in either pepper or tomato. Although a slight numerical tendency toward lower pH values was observed in the fertilized treatments, these results should be interpreted with caution. The application of nitrogen-based fertilizers leads to gradual soil acidification, an effect that may become more pronounced after long-term use of such inputs. Liqiang Zhang et al. (2024) explained this phenomenon by the fact that nitrogen transformations in soil are not fully balanced. The nitrification of ammonium ions (NH_4_^+^) produces protons (H^+^). The authors reported that when nitrogen inputs exceed plant uptake capacity, part of the nitrate (NO_3_^−^) may be lost through denitrification or leaching, thereby reducing proton consumption. This imbalance leads to a net accumulation of protons in the soil and consequently to a decrease in pH [29]. Although no statistically significant pH changes were detected in the present study, these mechanisms may explain the numerical trends observed following fertilizer application.

**Figure 1:**
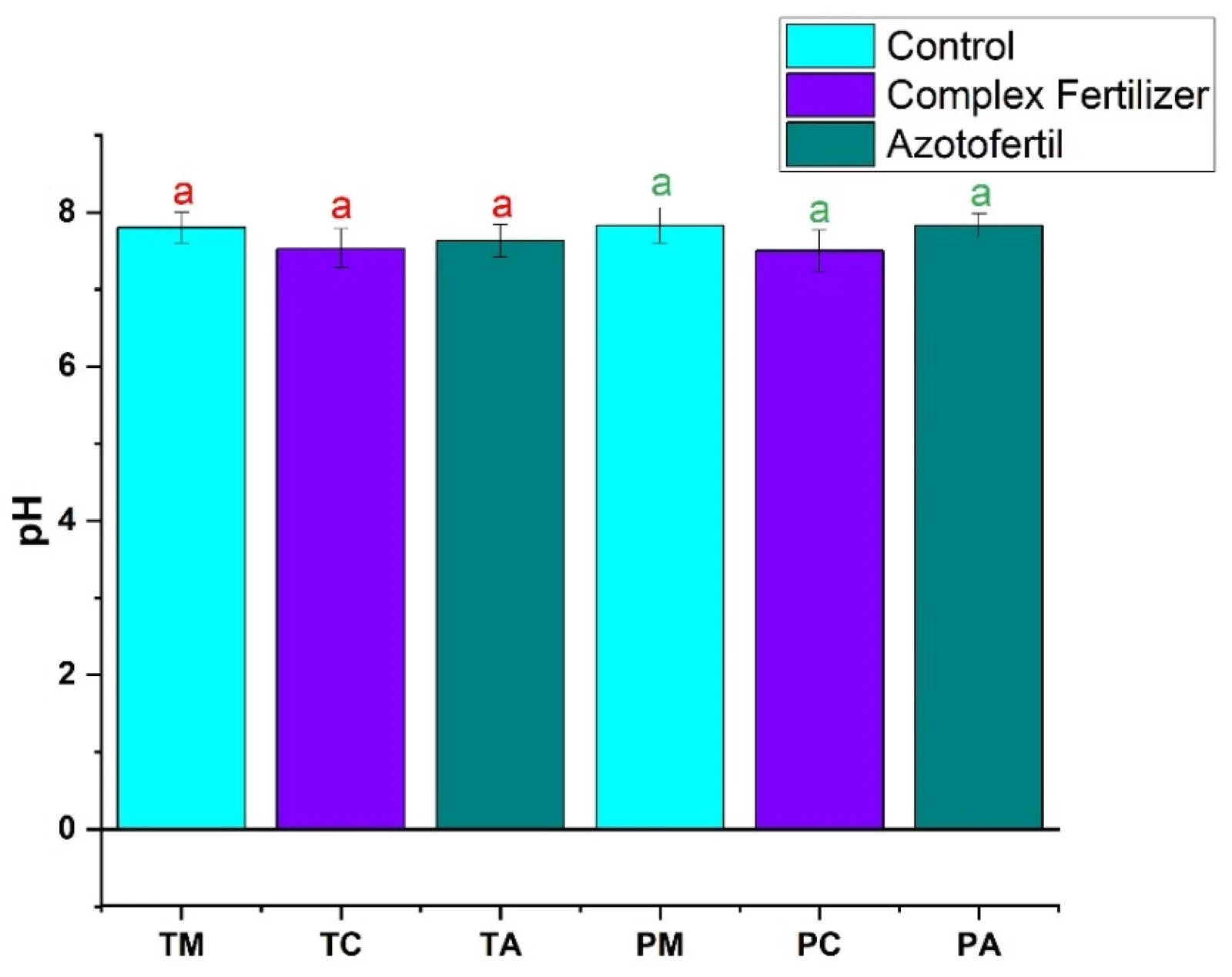
Effect of complex fertilizer and Azotofertil treatments on soil pH at the end of the experiment in tomato (TM, TC, TA) and pepper (PM, PC, PA) crops. TM and PM = untreated control; TC and PC = complex fertilizer treatment; TA and PA = Azotofertil treatment. Error bars represent standard deviation. Different letters indicate significant differences among treatments within each crop according to Duncan’s multiple range test (*p* < 0.05, n = 3).

The application of the complex fertilizer (based on nitrogen and phosphorus) was associated with a pH of 7.53 (3.5% lower than the untreated control), whereas the Azotofertil treatment showed a pH of 7.63 (Figure 1). Because these differences were not statistically significant, they should not be interpreted as evidence of a treatment effect. Nevertheless, the observed numerical trend is consistent with the expected response of soils receiving mineral nitrogen fertilization. The slightly higher pH recorded in the Azotofertil treatment may be related to the gradual release of plant-available nitrogen through biological nitrogen fixation, potentially reducing nitrogen losses by leaching, although this hypothesis requires further investigation.

In the pepper crop, a similar numerical trend was observed. The complex fertilizer showed a lower pH value (7.50) than the control (7.83), whereas the Azotofertil treatment displayed a pH value comparable to that of the control. However, these differences were also not statistically significant. Soil acidification is a well-documented consequence of long-term nitrogen fertilization. Xinqing Lu et al. (2023) reported that fertilizer-derived nitrogen is responsible for approximately 70% of soil pH decline in major crops such as wheat, rice, and maize, and up to 90% in fruit and vegetable crops [30]. Therefore, although the present results do not demonstrate significant short-term changes in soil pH, the observed numerical patterns are in agreement with mechanisms reported in the literature.

### 2.2. Determination of Cultivable Azotobacter spp.

**Figure 2:**
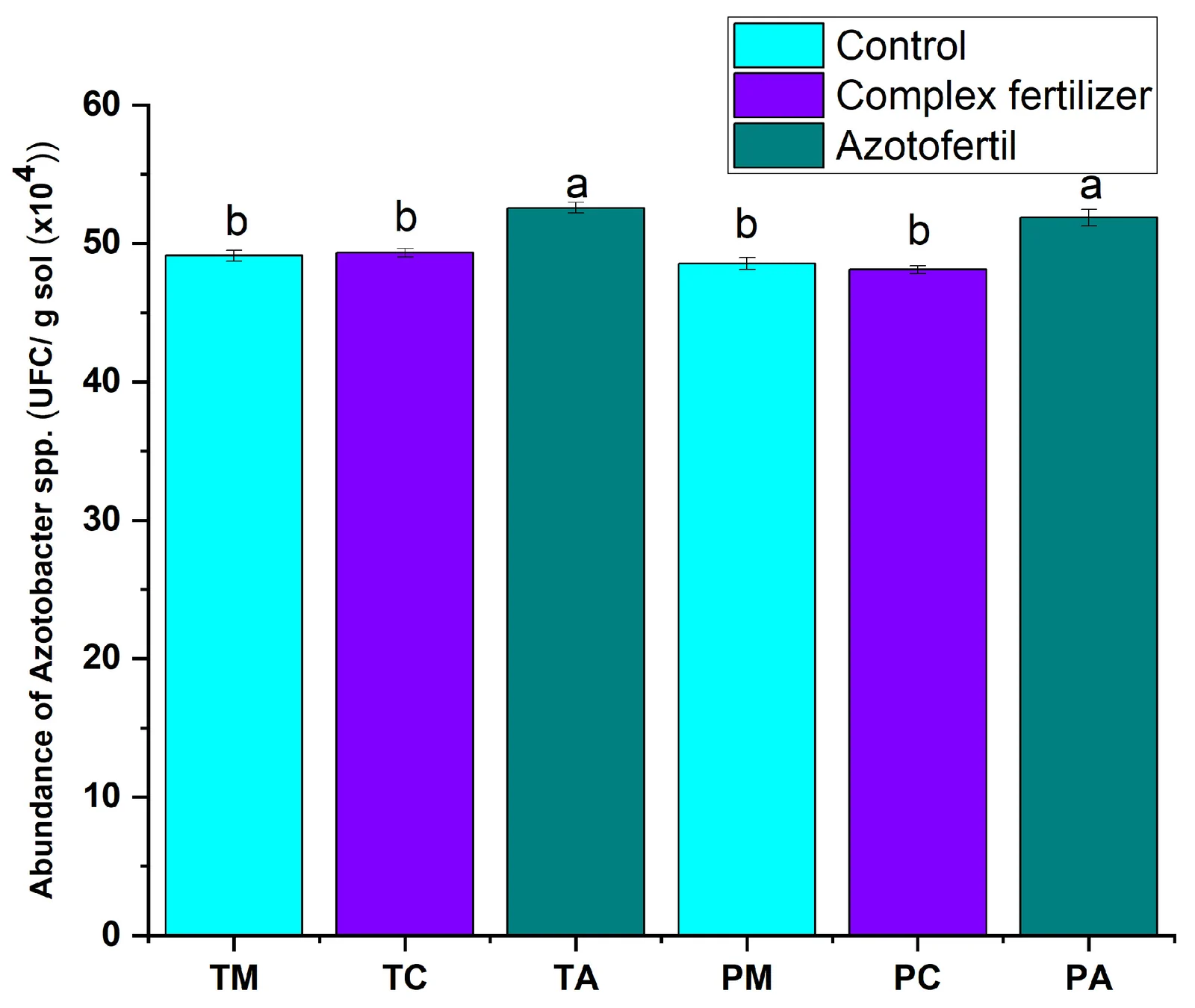
Effect of complex fertilizer and Azotofertil treatments on the abundance of *Azotobacter* spp. In soil at the end of the experiment in tomato (TM, TC, TA) and pepper (PM, PC, PA) crops. TM and PM = untreated control; TC and PC = complex fertilizer treatment; TA and PA = Azotofertil treatment. Error bars represent standard deviation. Different letters indicate significant differences among treatments within each crop according to Duncan’s multiple range test (*p* < 0.05, n = 3).

The application of the Azotofertil biostimulant significantly influenced the abundance of cultivable *Azotobacter* spp. populations in the soil of tomato and pepper crops (Figure 2). In both crops, the highest values were recorded in the Azotofertil-treated variant, reaching 52.57 × 10^4^ CFU g^−1^ soil in tomato and 51.88 × 10^4^ CFU g^−1^ soil in pepper. These values were significantly higher than those observed in both the control and the conventional complex fertilizer treatments, whereas no statistically significant differences were detected between the latter two treatments (Figure 2).

The increased abundance of *Azotobacter* spp. populations following Azotofertil application indicates that the treatment promoted the development of these beneficial bacterial populations under the experimental soil conditions. Similar findings were reported by Pandey and Maheshwari, who demonstrated that inoculated *Azotobacter chroococcum* strains were able to survive, proliferate, and effectively colonize the wheat rhizosphere. Likewise, Cirillo et al. showed that inoculation with *Azotobacter*-based microbial biostimulants increased rhizosphere microbial populations and enhanced soil biological activity [31,32]. The results obtained in the present study are consistent with these observations and indicate that Azotofertil application may promote the development of beneficial *Azotobacter* populations in the soil, thereby contributing to the improvement of the soil microbiological environment.

### 2.3. Plant Height Determination

The analysis of plant height showed numerically higher mean values in the fertilized treatments compared with the control. However, these differences were not statistically significant in either tomato or pepper. In both tomato and pepper, the highest mean plant height values were observed following the application of the complex fertilizer. In tomato plants, the mean plant height was 193 cm in the complex fertilizer treatment, compared with 176.5 cm in the control. Although this represented a numerical increase, the difference was not statistically significant.

A similar response has been reported in the literature. Andrew Jones et al. (2025) observed a slight vegetative increase in cabbage under a high nitrogen dose, although the differences were also not statistically significant. The authors attribute this observation to the plant’s nitrogen uptake pattern [33].

**Figure 3:**
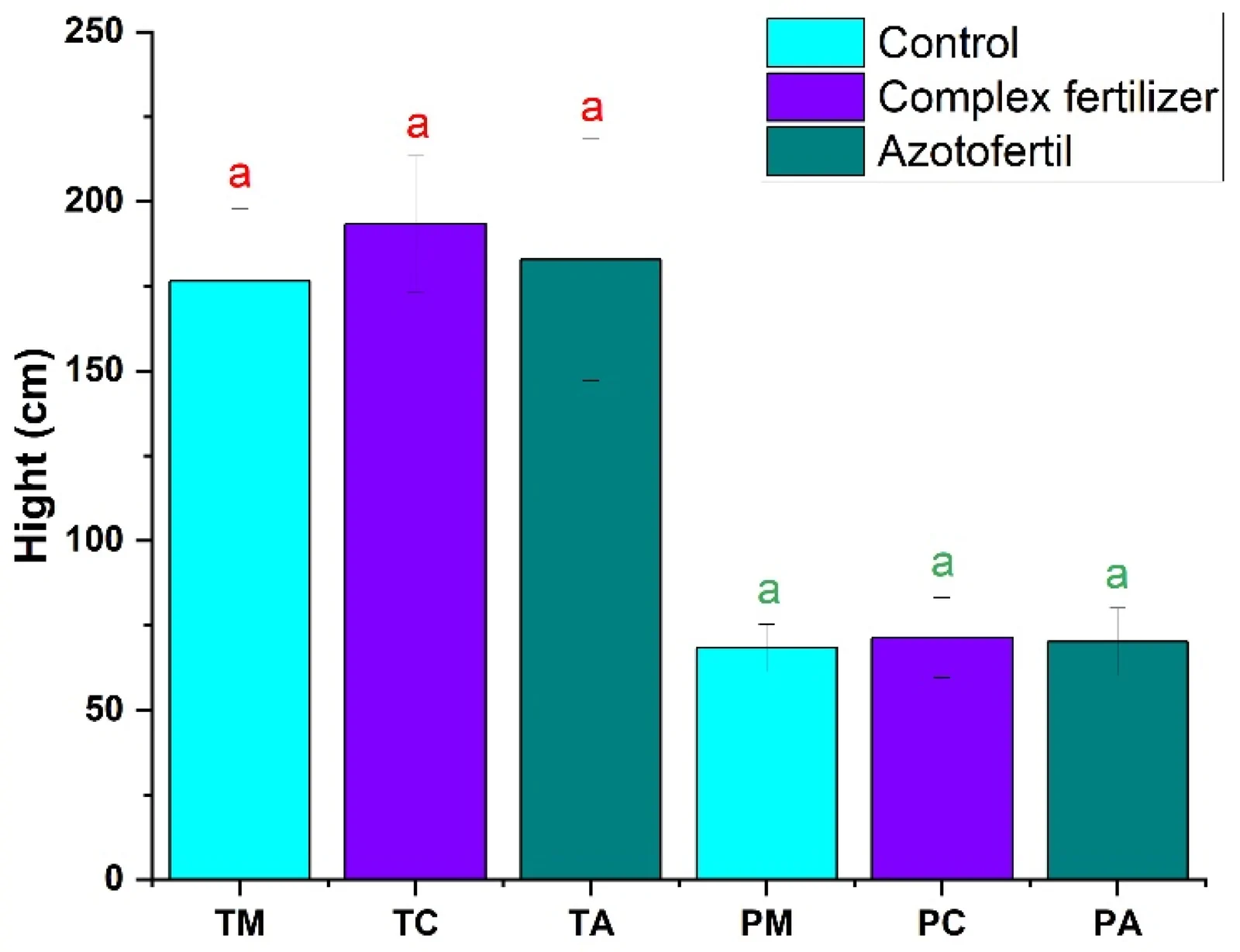
Effect of complex fertilizer and Azotofertil treatments on plant height in tomato (TM, TC, TA) and pepper (PM, PC, PA) plants. TM and PM = untreated control; TC and PC = complex fertilizer treatment; TA and PA = Azotofertil treatment. Error bars represent standard deviation. Different letters indicate significant differences among treatments within each crop according to Duncan’s multiple range test (*p* < 0.05, n = 3).

The Azotofertil treatment also showed slightly higher mean plant height values than the control; however, these differences were likewise not statistically significant (Figure 3). Therefore, the observed numerical trends should be interpreted with caution and cannot be considered evidence of a significant treatment effect under the conditions of the present study.

### 2.4. Determination of Total Chlorophyll Content

**Figure 4:**
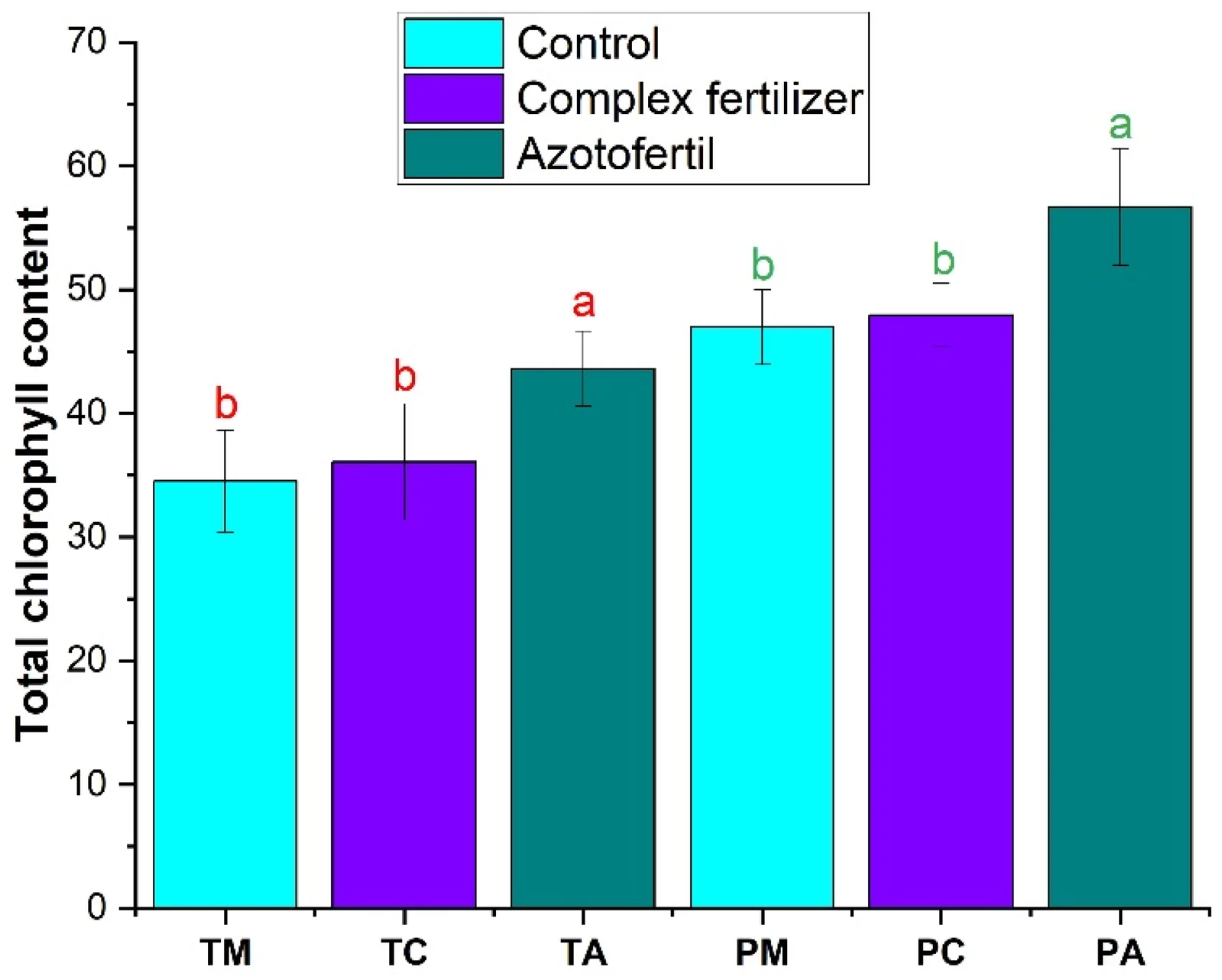
Effect of complex fertilizer and Azotofertil treatments on total chlorophyll content, expressed as SPAD units (s.u), in tomato (TM, TC, TA) and pepper (PM, PC, PA) plants. TM and PM = untreated control; TC and PC = complex fertilizer treatment; TA and PA = Azotofertil treatment. Error bars represent standard deviation. Different letters indicate significant differences among treatments within each crop according to Duncan’s multiple range test (*p* < 0.05, n = 3).

The use of SPAD analysis is supported by its rapid and non-destructive nature, as well as by the correlation between chlorophyll content and leaf nitrogen content, making it a reliable indirect indicator of the nitrogen nutritional status of plants [34]. In terms of total photosynthetic pigment content, an increasing trend was observed, particularly in the treatment with the nitrogen-fixing bacterial biostimulant. In tomato plants, an increase of 26% was recorded (from 34.5 s.u in the control to 43.5 in treated plants), while in pepper plants an increase of 21% was observed (from 46.97 s.u in the control to 56.68 s.u in treated plants) (Figure 4). The increases observed in plants treated with the complex fertilizer were not statistically significant compared to the control.

In addition to biological nitrogen fixation, the genus *Azotobacter* synthesizes and secretes considerable amounts of bioactive compounds such as B-complex vitamins, pantothenic acid, nicotinic acid, biotin, heteroauxins, gibberellins, and auxins [20]. Among these bioactive compounds, B-complex vitamins act as cofactors of enzymes involved in energy and photosynthetic metabolism, optimizing photosynthetic activity and electron transfer in biochemical reactions within chloroplasts [35,36]. Auxins increase the photosynthetic rate through chloroplast development, leaf expansion, and stomatal regulation, while gibberellins contribute through leaf growth, increased chlorophyll content, and enhanced activity of enzymes involved in carbon fixation and sugar metabolism [37]. It can thus be concluded that the high photosynthetic rate, reflected by the increased content of photosynthetic pigments, is the result of a combination of favorable factors induced by the activity of nitrogen-fixing bacteria.

### 2.5. Determination of Vitamin C

**Figure 5:**
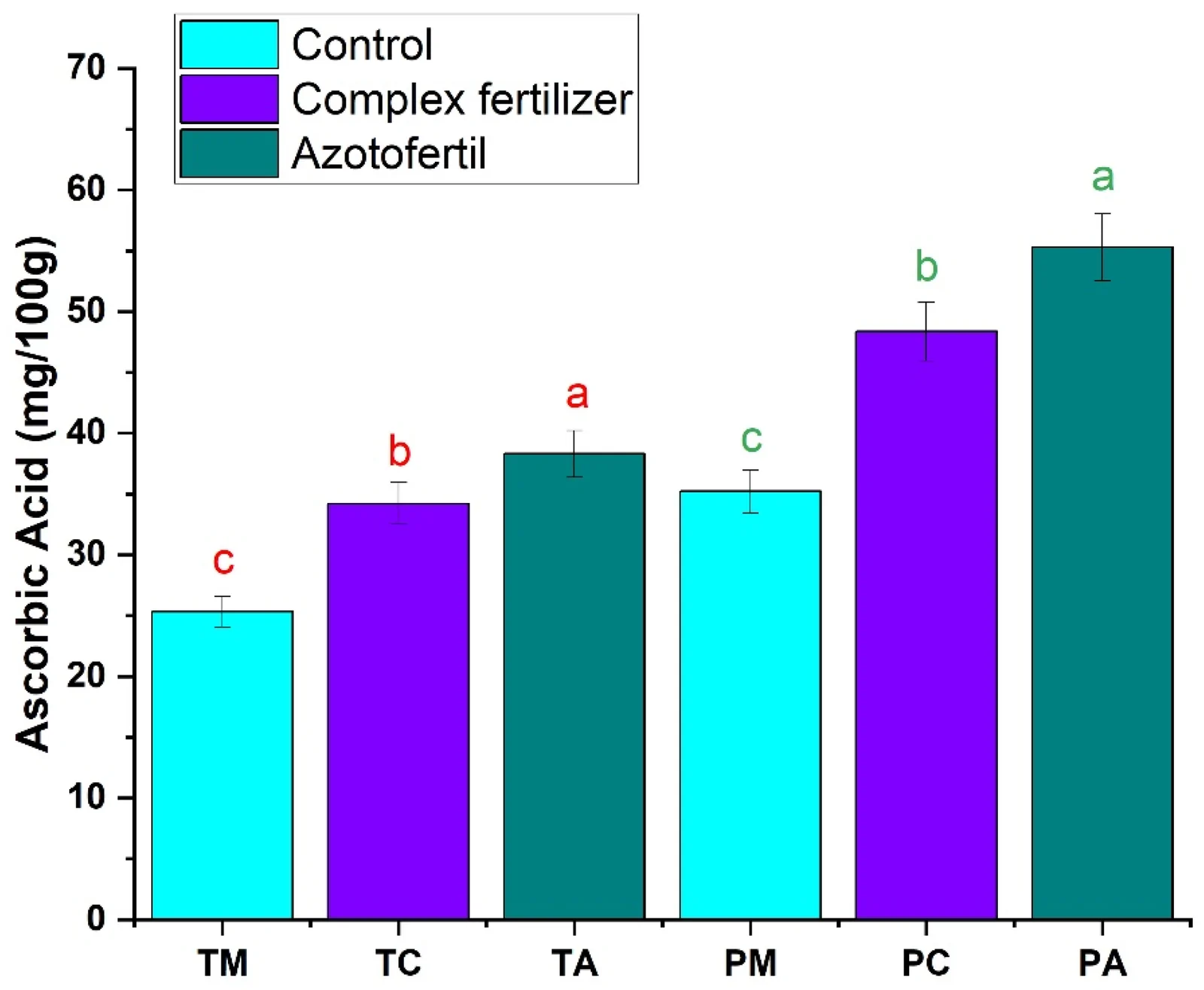
Effect of complex fertilizer and Azotofertil treatments on ascorbic acid content in tomato (TM, TC, TA) and pepper (PM, PC, PA) fruits, expressed as mg 100 g^−1^ fresh weight. TM and PM = untreated control; TC and PC = complex fertilizer treatment; TA and PA = Azotofertil treatment. Error bars represent standard deviation. Different letters indicate significant differences among treatments within each crop according to Duncan’s multiple range test (*p* < 0.05, n = 3).

Vitamin C content in tomato and pepper fruits increased following the application of both types of fertilizer treatments. In tomatoes, the complex fertilizer treatment resulted in a 35% higher ascorbic acid content compared to the control, while Azotofertil induced a 50% increase relative to the untreated control. In peppers, vitamin C content increased by 37% under the complex fertilizer treatment, whereas Azotofertil treatment resulted in a 57% increase compared with the untreated control (Figure 5).

The highest vitamin C content was observed under the nitrogen-fixing bacterial treatment. This response may be associated with the activity of the inoculated microorganisms, which are known to contribute to plant nutrition and metabolism through biological nitrogen fixation and the production of bioactive compounds. Moreover, the higher vitamin C content observed under this treatment may also be related to the maintenance of a higher photosynthetic capacity, which could favor glucose synthesis, an essential precursor in ascorbic acid biosynthesis [38]. Thiamine and nicotinic acid play important roles in energy metabolism, with NADPH being directly involved in the reduction in L-gulonolactone, the final step in vitamin C biosynthesis [39].

Similar responses were reported by Ordookhani et al. (2013), who inoculated tomato plants with PGPR, including *Azospirillum lipoferum*, and observed an increase in vitamin C content compared to the control. The authors specifically evaluated vitamin C and concluded that all microbial treatments improved fruit quality parameters [40].

### 2.6. Determination of Total Yield

Total tomato yield varied from 103.75 t/ha in the control variant to 135.79 t/ha in the variant fertilized with the phosphorus- and nitrogen-based complex fertilizer. The fertilization treatments applied to the tomato crop had a positive influence on total yield. In the variant treated with Azotofertil, an increase of 17.69 t/ha compared to the control was recorded, while in the variant treated with nitrogen and phosphorus, increases of 32.05 t/ha compared to the control were observed.

**Figure 6:**
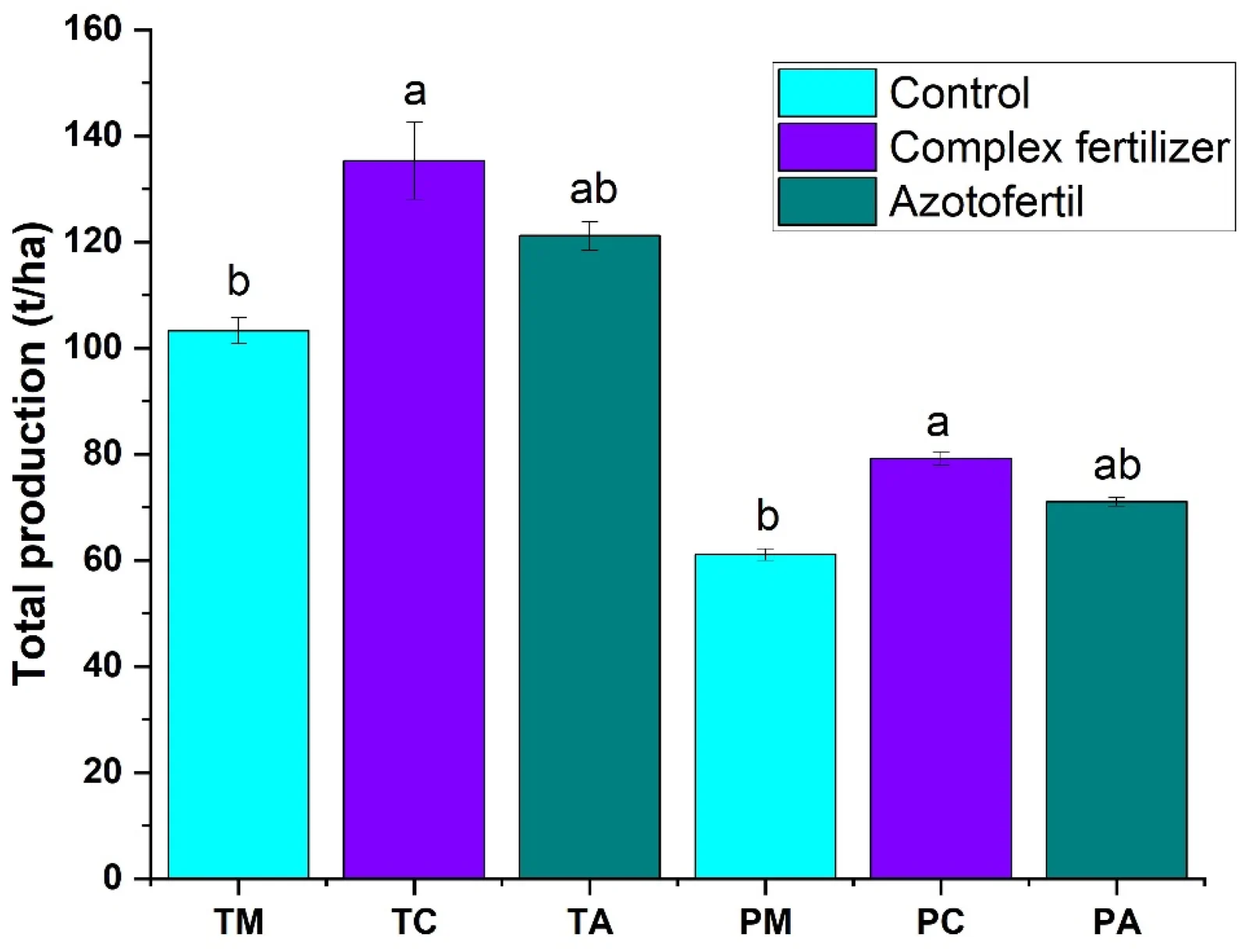
Effect of complex fertilizer and Azotofertil treatments on total yield in tomato (TM, TC, TA) and pepper (PM, PC, PA) crops, expressed as t ha^−1^. TM and PM = untreated control; TC and PC = complex fertilizer treatment; TA and PA = Azotofertil treatment. Error bars represent standard deviation. Different letters indicate significant differences among treatments within each crop according to Duncan’s multiple range test (*p* < 0.05, n = 3).

Total pepper yield ranged from 61.20 t/ha in the control variant to 79.60 t/ha in the variant fertilized with the complex fertilizer (Figure 6). As in tomato, fertilization treatments improved total pepper yield. Under Azotofertil treatment, an increase of 9.85 t/ha compared to the control was observed, while in the nitrogen and phosphorus treatment, total yield increased by 18.41 t/ha compared to the control.

The higher yield recorded under the conventional fertilizer treatment may be associated with the combined supply of nitrogen and phosphorus, both of which play essential roles in plant growth, flowering, and fruit development [41,42,43]. This observation is consistent with the established roles of these nutrients in supporting crop productivity.

Thus, although the nitrogen-fixing bacterial biostimulant does not produce the same positive effect on yield, a considerable improvement compared to the control is still observed under conditions of reduced nitrogen input from conventional fertilization methods.

Pearson correlation analysis was conducted as an exploratory analysis to visualize potential relationships among the analyzed parameters. Thus, the intensity and direction of the relationships between soil pH and total yield, photosynthetic pigment content and vitamin C content, as well as plant height and total yield, were investigated.

For the construction of the correlation matrices, specific notations were used, including the analyzed parameters (pH, TP—total yield, H—plant height, Chl—chlorophyll pigments, and VC—vitamin C), the studied species (T—tomato and P—pepper), and the applied treatment (M—control, C—complex fertilizer, and A—Azotofertil). The correlation matrices are displayed using a color scale depending on the degree of correlation between parameters: a strong negative correlation (r = −1) is represented in dark blue, no correlation (r = 0) in gray, and a strong positive correlation (r = 1) in yellow. Since color variations may be difficult to perceive, the matrix also includes the actual correlation coefficient values in the upper part of the heatmap. Because the analysis was performed using three biological replicates per treatment, the correlation coefficients were interpreted as exploratory indicators of potential relationships among the analyzed parameters, with particular caution in the case of very high correlation values.

**Figure 7:**
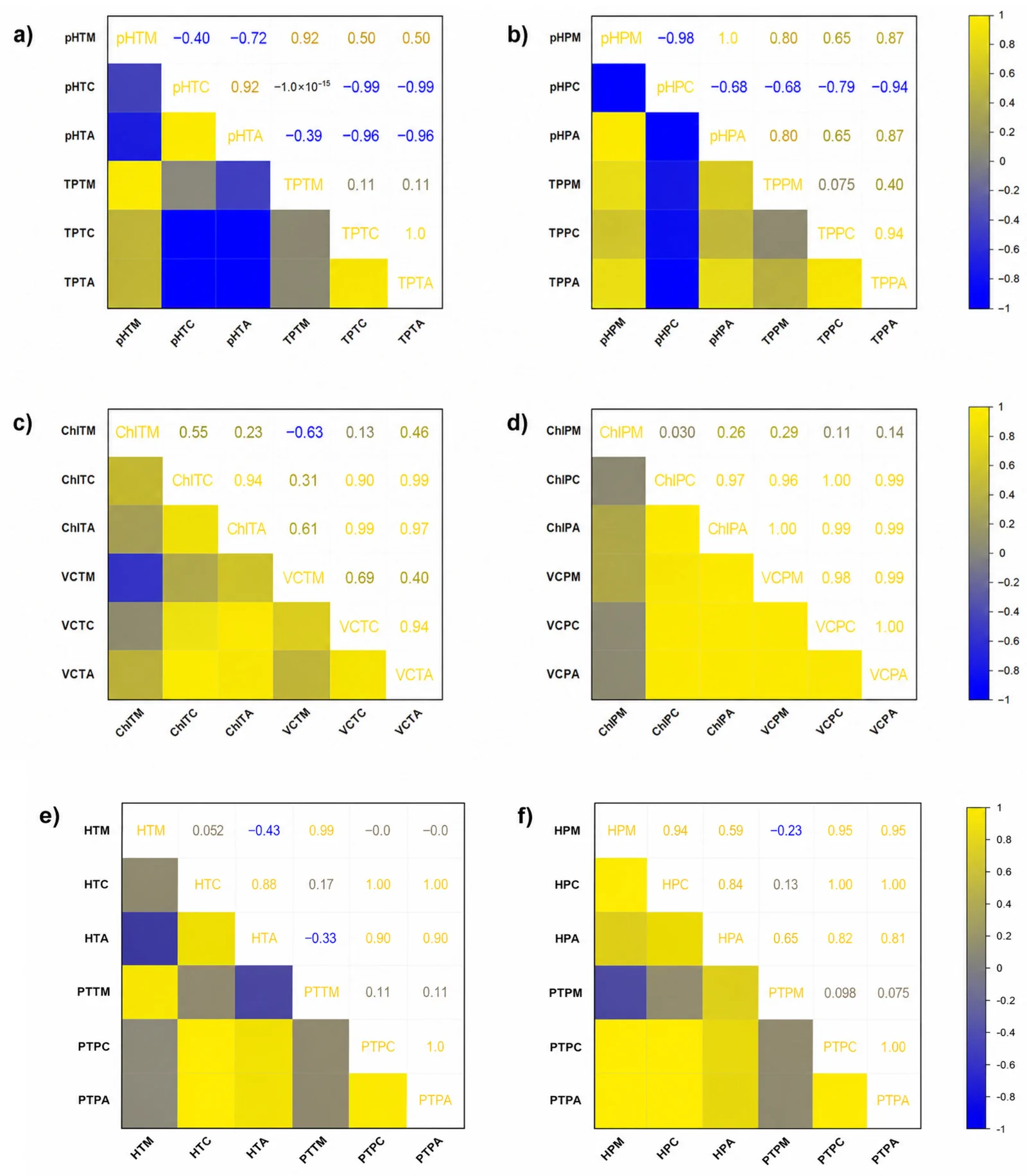
Pearson correlation matrices for: (**a**) soil pH and total yield in tomato; (**b**) soil pH and total yield in pepper; (**c**) chlorophyll and vitamin C in tomato; (**d**) chlorophyll and vitamin C in pepper; (**e**) plant height and total yield in tomato; (**f**) plant height and total yield in pepper.

In tomato plants, the Pearson correlation between soil pH and yield (Figure 7a) showed a negative association under both the complex fertilizer and Azotofertil treatments, representing an exploratory pattern in which lower soil pH values tended to coincide with higher yields under the experimental conditions. A similar trend was observed in pepper plants under the complex fertilizer treatment (Figure 7b), whereas this negative association was less evident following Azotofertil application. A strong positive correlation was observed between pH_TM and pH_TA, representing an exploratory similarity between the measured soil pH values recorded in the control and Azotofertil treatments.

The Pearson correlation between vitamin C and chlorophyll pigments (Figure 7c) revealed a strong positive association in tomato plants. The highest correlation coefficient was observed under the Azotofertil treatment, where Chl_TA and VC_TA showed *r* = 0.97, representing an exploratory positive association between chlorophyll pigment content and vitamin C under the experimental conditions. A similar trend was observed in pepper plants (Figure 7d).

Regarding the Pearson correlation between plant height and total yield, the strongest positive association was observed under the complex fertilizer treatment (TP_TC and H_TC, *r* = 1) (Figure 7e), representing a pattern of covariation between plant height and total yield under this treatment. High positive correlations were also observed following Azotofertil application (*r* = 0.90), indicating a similar positive association between plant height and total yield. The same pattern was observed in pepper plants, where similar exploratory correlation patterns between vegetative growth and yield were also observed.

Overall, the observed Pearson correlations suggest potential biological relationships among the analyzed parameters. However, because the analyses were based on only three biological replicates per treatment, the correlation coefficients should be interpreted with caution and regarded as exploratory indicators of potential associations rather than conclusive evidence.

## 3. Materials and Methods

### 3.1. Materials

The materials used in the present study included the tomato hybrid *Cristal F1* (*Solanum lycopersicum* L.), the sweet pepper hybrid *Piedone F1* (*Capsicum annuum* L.), the biofertilizer Azotofertil, a commercial mineral fertilizer, ferric chloride (FeCl_3_), potassium ferricyanide (K_3_[Fe(CN)_6_]), potassium chloride (KCl), hydrochloric acid (HCl), nitric acid (HNO_3_), mannitol, dipotassium phosphate (K_2_HPO_4_), magnesium sulfate heptahydrate (MgSO_4_·7H_2_O), sodium chloride (NaCl), potassium sulfate (K_2_SO_4_), calcium carbonate (CaCO_3_), and bacteriological agar.

**Figure 8:**
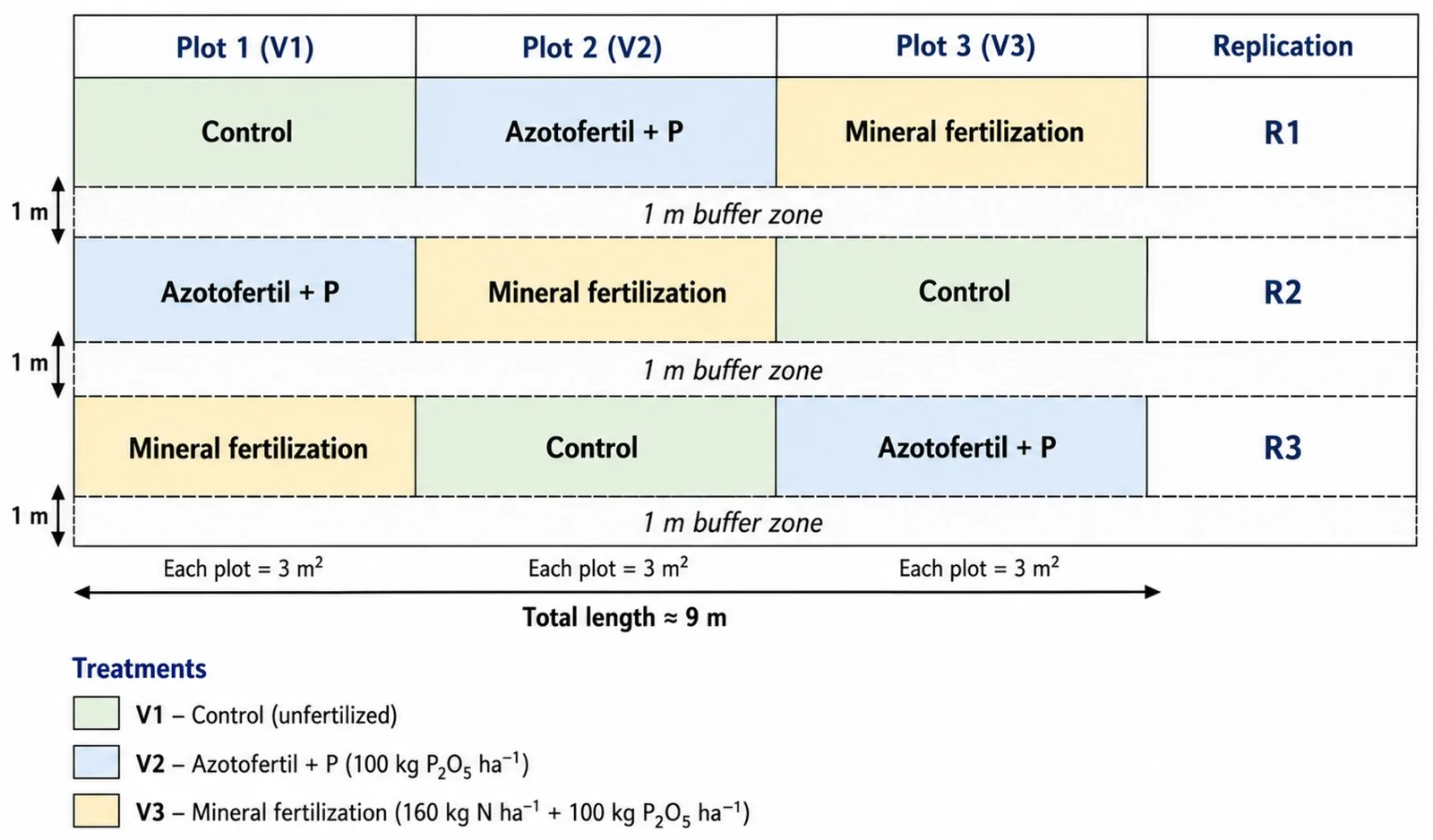
Schematic representation of the field experimental design.

The biofertilizer used in this study was Azotofertil (S.C. Antibiotice S.A., Iași, Romania), formulated as a liquid suspension containing the nitrogen-fixing bacteria *Azotobacter chroococcum* and *Azospirillum lipoferum*. According to the manufacturer’s specifications, these microorganisms are capable of biologically fixing atmospheric nitrogen and colonizing the plant root system. Mineral fertilization was performed using a commercial fertilizer to provide a total of 160 kg N ha^−1^ and 100 kg P_2_O_5_ ha^−1^, according to the experimental design (Figure 8).

### 3.2. Experimental Design

The experiment was conducted at the “Vasile Adamache” Research and Student Practice Station of the Ion Ionescu de la Brad University of Life Sciences, Iași, Romania, from April to August 2024. The experimental field is characterized by an aric-cambic chernozem with a loamy-clay texture, a soil type typical of the agricultural area surrounding Iași and considered highly suitable for horticultural crop production [44]. The study comprised two independent experiments conducted separately for tomato and sweet pepper, using the same experimental scheme. For each crop, a one-factor experimental design was used, with fertilization treatment as the experimental factor. Each experiment was arranged in a randomized complete block design (RCBD) and included three experimental treatments, with three biological replicates per treatment. Each experimental plot consisted of 12 plants. plants. To minimize the effects of spatial variability and prevent interference among treatments, adjacent plots were separated by 1 m buffer zones (Figure 8). The following experimental treatments were evaluated:

V1 (Control): unfertilized control;

V2 (Azotofertil + P): phosphorus fertilization supplemented with the Azotofertil biofertilizer;

V3 (Mineral fertilization): mineral fertilization with nitrogen (N) and phosphorus (P_2_O_5_).

### 3.3. Treatment Application

Treatments were applied according to the experimental protocol established for each crop. The control treatment received no fertilization. In the V2 (Azotofertil + P) treatment, phosphorus was applied in two split applications: 60 kg P_2_O_5_ ha^−1^ during seedbed preparation and 40 kg P_2_O_5_ ha^−1^ during the growing season. The Azotofertil biofertilizer was applied at a rate of 5 L ha^−1^ during seedbed preparation and an additional 5 L ha^−1^ during the growing season, when the fruit of the first inflorescence reached approximately 1 cm in diameter. In the V3 (Mineral fertilization) treatment, 60 kg N ha^−1^ and 60 kg P_2_O_5_ ha^−1^ were applied during seedbed preparation, followed by 100 kg N ha^−1^ and 40 kg P_2_O_5_ ha^−1^ during the growing season. All physiological and biochemical determinations were performed 20 days after the application of the second fertilization treatment, during the fruiting stage. This sampling time was selected to evaluate the final physiological response of the plants following completion of the fertilization program, as well as its potential effects on fruit quality.

### 3.4. Soil pH Determination

Soil samples were air-dried at room temperature or oven-dried at a temperature not exceeding 40 °C. For each crop species, three soil pH analyses were performed for each experimental treatment (Control, V2, and V3). After removing plant residues and coarse materials, the samples were crushed, homogenized, and passed through a 2 mm sieve. Soil pH was determined using the potentiometric method in a 1:2.5 (*w*/*v*) soil-to-water suspension. For each determination, 10 g of soil were mixed with 25 mL of distilled water. The suspension was stirred for approximately 10 min and allowed to equilibrate for 2 h. Soil pH was measured using a SevenCompact S220 pH meter (Mettler-Toledo, Greifensee, Switzerland), calibrated prior to the measurements. The results were expressed as pH units and calculated as the mean of two readings differing by no more than 0.05 pH units [44].

### 3.5. Determination of Cultivable Azotobacter spp.

Soil samples were collected from a depth of 10 cm using a sterile metal soil probe and transported to the laboratory in paper bags. For each crop species, soil samples were collected from three biological replicates of each experimental treatment (Control, V2, and V3). The samples were crushed and homogenized in a sterile mortar. One gram of soil from each sample was weighed, and successive decimal dilutions were prepared up to 10^−3^ and 10^−4^. From each dilution, 1 mL of the suspension was transferred into a Petri dish, and the selective culture medium, previously melted and cooled to approximately 45 °C, was added. The inoculum was evenly distributed by gently rotating the plates before the medium solidified. Three plates were prepared for each dilution. The plates were incubated at 28 °C for 24 h, after which bacterial colonies were counted. The results were expressed as colony-forming units per gram of soil (CFU g^−1^ soil).

### 3.6. Plant Height Determination

Plant height was measured using a graduated measuring tape by determining the distance between the soil surface and the uppermost point of the main stem. Nine plants were evaluated for each experimental treatment. Measurements were performed after the application of the final treatment, during the fruiting stage. The results were expressed in centimeters (cm).

### 3.7. Chlorophyll Content

The leaf chlorophyll content index was determined non-destructively using a SPAD-502 Plus chlorophyll meter (Konica Minolta, Tokyo, Japan). Nine measurements were performed on fully expanded leaves for each experimental treatment, and the mean value was used for statistical analysis. The results were expressed in SPAD units (s.u).

### 3.8. Determination of Vitamin C Content

Ascorbic acid content was determined spectrophotometrically using the Prussian blue method, according to the procedure described by Ostaci et al. (2024). One gram of fresh fruit tissue was homogenized with 15 mL of 0.01 M HNO_3_. The homogenate was heated at 90 °C for 15 min, cooled to room temperature, and centrifuged at 2000 rpm for 5 min. One milliliter of the supernatant was diluted with 9 mL of 0.01 M HNO_3_. For color development, 1 mL of the diluted extract was mixed with 1 mL of 2 × 10^−3^ M FeCl_3_, 1 mL of 2 × 10^−3^ M K_3_[Fe(CN)_6_], 1 mL of 0.1 M KCl, and 1 mL of 0.01 M HCl. Absorbance was measured at 700 nm using a SPECORD 210 Plus spectrophotometer (Analytik Jena, Jena, Germany). Three analyses were performed for each experimental treatment (Control, V2, and V3), and the mean value was used for subsequent statistical analysis. Quantification was performed using a calibration curve prepared with L-ascorbic acid standard solutions obtained from a 1 g L^−1^ stock solution. Vitamin C content was calculated from the calibration curve over the concentration range of 1–10 mg L^−1^ (y = 0.00683x + 0.00299; R^2^ = 0.998) [45].

### 3.9. Determination of Total Yield

Total yield was determined by harvesting and weighing all fruits that reached harvest maturity from all plants within each experimental plot. Nine yield determinations were performed for each experimental treatment (Control, V2, and V3), and the mean value was used for subsequent statistical analysis. The fruits were weighed immediately after harvest, and the yield obtained for each plot was expressed on a hectare basis. Total yield was calculated based on the fresh weight of all marketable fruits harvested from each experimental plot and the corresponding harvested area, using the following equation:

$$Y\;\left(t\;{ha}^{-1}\right)=\;\frac{W\;(kg)}{A(m^{2})}\;\times \;\frac{10,000{\;m}^{2}\;{ha}^{-1}}{1000\;\mathrm{kg}\;t^{-1}}=\;\frac{W}{A}\;\times 10$$

where

*Y* represents total yield expressed in t ha^−1^,

*W* represents the total fresh fruit weight harvested from the experimental plot, expressed in kg,

*A* represents the harvested plot area, expressed in m^2^

The factor 10 results from converting kg m^−2^ to t ha^−1^ [46].

### 3.10. Statistical Analysis

All statistical analyses were performed using Microsoft Excel and OriginPro (OriginLab Corporation, Northampton, MA, USA). Experimental data were obtained from three biological replicates and are presented as mean ± standard deviation (SD).

Differences among treatments were evaluated by one-way analysis of variance (ANOVA), followed by Duncan’s multiple range test to identify statistically significant differences between treatment means at *p* < 0.05. Duncan’s multiple range test and graphical representations were performed using OriginPro.

Pearson correlation analysis was carried out using Microsoft Excel to evaluate the relationships between the analyzed parameters. Correlation matrices were visualized using OriginPro. For all statistical analyses, differences were considered statistically significant at *p* < 0.05. Data normality was assessed using online Kolmogorov–Smirnov test, while homogeneity of variances was evaluated using Levene’s test for equal variances in OriginPro 2021 (OriginLab Corporation, Northampton, MA, USA).

## 4. Conclusions

The application of the Azotofertil biostimulant increased the abundance of *Azotobacter* spp. in the soil, confirming its ability to promote the development of beneficial soil microorganisms associated with biological fertilization. This finding supports the potential of bacterial biostimulants as a component of sustainable nutrient management strategies.

The application of the Azotofertil biostimulant, based on the nitrogen-fixing bacteria *Azotobacter chroococcum* and *Azospirillum lipoferum*, had beneficial effects on *Solanum lycopersicum* L. and *Capsicum annuum* L., resulting in increased photosynthetic pigment content, vitamin C content, and total yield compared with the unfertilized control. The most pronounced effects were observed for quality-related parameters, with chlorophyll content increasing by 26% in tomato and 21% in pepper, while vitamin C content increased by 50% and 57%, respectively.

Although conventional mineral fertilization produced the highest yields, Azotofertil also resulted in substantial improvements compared with the unfertilized control. Therefore, bacterial biostimulants should not be regarded as a replacement for conventional fertilization but rather as a complementary strategy for improving crop performance. Their beneficial effects on physiological, quality-related, and yield parameters support their potential for integration into horticultural production systems and encourage further research under different cultivation conditions.

## Data Availability

Data are contained within the article.
